# Expression level of integrin *α*5 on tumour cells affects the rate of metastasis to the kidney

**DOI:** 10.1038/sj.bjc.6600710

**Published:** 2003-01-28

**Authors:** N Tani, S Higashiyama, N Kawaguchi, J Madarame, I Ota, Y Ito, Y Ohoka, S Shiosaka, Y Takada, N Matsuura

**Affiliations:** Division of Structural Cell Biology, Nara Institute of Science and Technology (NAIST), 8916-5 Takayama, Ikoma, Nara 630-0101, Japan; Department of Pathology, School of Allied Health Science, Faculty of Medicine, Osaka University, 1-7 Yamada-oka, Suita, Osaka 565-0817, Japan; Department of Vascular Biology, The Scripps Research Institute, 10550 North Torrey Pines Road, La Jolla, CA 92037, USA

**Keywords:** tumour metastasis, *α*5*β*1 integrin, kidney, CHO-K1

## Abstract

Tumour metastasis is known clinically to have organ specificity. We hypothesised that integrins might be involved in determining the organ specificity of tumour metastasis. Here, we report the results of spontaneous metastasis tested in nude mice that were inoculated with Chinese hamster ovary (CHO) cells expressing integrin *α*5*β*1 at various levels. The growth of the primary tumour inversely correlated with the *α*5 expression level on CHO cells, which is consistent with a previous report (Schreiner *et al*, 1991). The rates of pulmonary, lymph node, and adrenal metastases that developed in nude mice were not related to changes of the *α*5 expression level on CHO cells. Kidney metastasis developed in 40% of nude mice inoculated with *α*5B2 cells (CHO cells overexpressing *α*5) and in 20% of mice with CHO-K1 cells (CHO cells expressing native *α*5), whereas inoculation with CHO-B2 cells (*α*5-defective mutants) and *α*5CHO cells with the highest expression of *α*5 did not lead to development of kidney metastasis. Furthermore, *α*5CHO, which shows the slowest growth of these cell types, did not lead to primary tumours in nude mice. These findings suggest that there is an appropriate level of *α*5 expression on tumour cells that leads to metastasis. Microscopic observations revealed that micrometastasis in the kidney was formed in glomeruli. An adhesion assay using frozen sections of the kidney demonstrated that *α*5B2 cells, but not CHO-B2 cells, effectively adhered to glomeruli. Kidney metastasis *in vivo* and the adhesion of *α*5B2 to glomeruli shown *ex vivo* were significantly suppressed by the administration of GRGDS peptide. Finally, we conclude that the interaction of *α*5*β*1 on tumour cells with fibronectin in kidney glomeruli is involved in kidney metastasis and that the tumour has appropriate levels of integrins crucial for metastasis.

## Introduction

Clinically, it is well known that tumour metastasis has organ specificity. Selectivity of the organs to which the tumour metastasises is thought to be affected by interactions between the tumour and molecules surrounding it (seed and soil theory) (Paget, 1898), and the direction of blood flow (mechanical theory) (Ewing, 1928). The process of tumour metastasis consists of detachment of cells from the primary tumour, invasion of extracellular matrix (ECM), intra- and extravasation, and growth at the secondary site. Numerous data have been reported to support the seed and soil theory as well as the mechanical theory by means of these processes. In B16-F10 melanoma cells and reticulum-cell sarcoma cells, organ specificity in their binding *in vitro* and *ex vivo* reflected the organ specificity of their metastatic distribution *in vivo* (Nicolson and Winkelhake, 1975; Netland and Zetter, 1984). Tumour cells that metastasised selectively to a specific organ showed intense adhesion to the endothelial cells derived from this organ (Nicolson, 1988; Pauli and Lee, 1988). Furthermore, an adhesion molecule designated Lu-ECAM-1 is specifically expressed on endothelial cells derived from the lung but not from other organs (Zhu *et al*, 1991). These findings suggest that there might be specific molecules or proportions of proteins in endothelial cells or ECM proteins in organs, that determine the metastatic sites of tumour cells. Therefore, adhesion molecules appear to be crucial for mediating the tumour–endothelial or tumour–ECM interactions.

Integrins are transmembrane glycoproteins that consist of *α* and *β* subunits and mediate cell–matrix and cell–cell adhesions (Hynes, 1992). Varieties of *α* and *β* subunits produce ligand selectivity to ECM (Albelda and Buck, 1990). To elucidate the role of integrins in tumour progression leading to metastasis, integrin expression levels have been compared in normal and tumour cells, or in primary and secondary sites in cancer patients using polymerase chain reaction (PCR), *in situ* hybridization, or immunohistochemical techniques.

Changes of integrin expression or its localisation in transformed cells appear to contribute either positively or negatively to the transformed cell phenotype. For instance, *α*5*β*1 integrin is a key molecule in the invasion at least of melanoma, osteosarcoma, and glioblastoma cells, while *α*V*β*3 integrin also appears to be associated with increased invasiveness (Ruoslahti, 1992). The localisation of the fibronectin receptor on the cell surface is altered in the malignant transformation of cultured human cells in comparison with normal cells (Akiyama *et al*, 1990). In sarcoma virus transformation of several rodent cell lines, *α*5*β*1 integrin disappears from the cell surface, whereas *α*3*β*1 levels remain constant (Heino, 1993). In melanoma, alterations of *α*2*β*1, *α*3*β*1, and *α*6*β*1 integrins appear to be associated with malignancy (Chen *et al*, 1991; Natali *et al*, 1991, 1993; Moretti *et al*, 1993). In breast cancer, *α*2*β*1, *α*5*β*1, or *α*6*β*4 integrins seem to be crucial for malignant transformation (Pignatelli *et al*, 1991; Natali *et al*, 1992; Zutter *et al*, 1993; Weaver *et al*, 1997). In pancreatic carcinoma cell lines, expression of *α*2, *α*3, *α*6, *β*1, *β*4, and *β*5 integrins was detected in adenocarcinomas and ampullary tumours, in the normal pancreas, reduced levels of expression occur, or in the case of some integrins there is no expression (Hall *et al*, 1991). These strategies are beneficial in that integrin expression is directly detected on tissues from patients or tumour cell lines.

In another strategy, tumour cells or mice with genetic changes of integrin expression have been used for analysing the effects of integrin on tumourigenesis and organ-preferential metastasis. Rhabdomyosarcoma (RD) cells transfected with cDNA encoding the *α*2 subunit enhanced metastasis in nude mice, demonstrating the specific effect of *α*2*β*1 integrin on metastasis (Chan *et al*, 1991). CHO cells transfected with *α*4 cDNA caused experimental bone metastasis in nude mice, suggesting that bone metastasis was induced by interactions between *α*4*β*1 on tumour cells and VCAM-1 on stromal cells in the bone marrow (Matsuura *et al*, 1996). Here, we constructed CHO transfectants expressing *α*5*β*1 at various levels and examined metastatic sites in nude mice. In this study, we especially focused on the relation between the change in *α*5*β*1 levels on tumour cells and rates of metastasis.

## MATERIALS AND METHODS

### Cells and culture conditions

CHO-K1 cells (ATCC, VA, USA) were used as parent cells in this study. CHO-B2 cells are *α*5-defective mutants of CHO-K1 cells, that exclusively express *α*5*β*1 integrin (Schreiner *et al*, 1989), and their transfectants were designated as *α*5B2 and *α*5CHO cells, respectively. A pBJ-1 vector carrying *α*5 cDNA driven by the SR*α* promoter (Takebe *et al*, 1988) was transfected into CHO-K1 or CHO-B2 cells. Cells expressing *α*5 were selected by flow cytometric sorting and by resistance to geneticin (G418). *α*5B2F187A cells are transfectants of CHO-B2 cells with mutant *α*5 cDNA containing an alanine substitution of phenylalanine 187 (Irie *et al*, 1995). Cells were maintained in Dulbecco's modified Eagle's medium (DMEM, Nihonseiyaku, Tokyo, Japan), supplemented with 10% fetal bovine serum (FBS, Dainippon Pharmaceutical Co., Ltd, Tokyo, Japan) and 50 U ml^−1^ of penicillin plus 50 *μ*g ml^−1^ of streptomycin (Life Technologies, Frederick, ML, USA) in 10% CO_2_.

### Flow cytometric analysis

Cells in DMEM supplemented with 1% FBS and 0.03% sodium azide were incubated with a monoclonal antibody against human/hamster *α*5 (KH72) for 30 min at 4°C. After washing with DMEM as described above, the cells were incubated with FITC-conjugated mouse IgG (DAKO, Copenhagen, Denmark) for 30 min at 4°C. After washing, cells were resuspended with the same DMEM and analysed by FACS Calibur (BD Biosciences, CA, USA).

### Spontaneous metastasis model in nude mice

Nude mice (BALB/cAnNCrj-nu/nu, 4 weeks, female) were subcutaneously injected with 1×10^6^ cells of parental, mutant, or transfectant CHO cells. Subcutaneous tumours were measured with a dial-caliper twice a week and volumes were determined using the formula width^2^×length×0.52. Each volume value indicates the mean±standard errors (s.e.). Primary tumours were removed surgically at a size of 2500–3500 mm^3^ to prevent mice from dying. The mice were killed and metastatic sites were examined 7–8 weeks after inoculation. To investigate whether kidney metastasis was caused by adhesion of *α*5*β*1 on tumour cells to fibronectin, the effects of intravenous tail vein injection of GRGDS or GRGES peptide (20 mg kg^−1^ in 50 *μ*l each) in PBS had been tested every other day starting from day 5 for 7 weeks.

### Histological analysis

Kidney and lung were excised and fixed with formalin. Horizontal sections (6 *μ*m thick) were prepared from the centre of the tumour in paraffin-embedded tissue. The sections were stained with haematoxylin–eosin and metastatic sites were examined by microscopy.

### Adhesion assay

A total of 96-well plastic plates were coated with 10 *μ*g ml^−1^ of fibronectin in phosphate-buffered saline (PBS) for 2 h at 37°C and then treated with 3% bovine serum albumin (BSA) for 1 h at 37°C, or were coated with only BSA for negative control. The cells (5×10^4^ cells well^−1^) in serum-free DMEM containing 0.1% BSA were added and incubated for 1 h at 37°C. After removal of the medium, a 0.04% crystal violet solution was added and incubation was conducted for 10 min at room temperature. The wells were washed three times with PBS and 20 *μ*l of Triton X-100 was added for permeabilisation. After addition of distilled water to 100 *μ*l, the optical density was measured at 550 nm. The value indicates the mean±s.e. for triplicate data representing three independent experiments.

### Spreading assay

Cells (5×10^3^ cells well^−1^) in serum-free DMEM containing 0.1% BSA were added to plates coated with 10 *μ*g ml^−1^ of fibronectin (96 wells), and incubated for 3 h at 37°C. The cells were photographed on slide film under microscopy and the images on the film were analysed by NIH Image (National Institutes of Health, Bethesda, MD, USA). The spreading area of each cell type was calculated. The value indicates the mean±s.e. for 20 cells randomly sampled.

### Migration assay

Cell migration to soluble fibronectin was assessed by multiwell Boyden chambers (Neuro Probe, Gaithersburg, MD, USA). Polycarbonate filters (pore size of 8 *μ*m) were coated with 10 *μ*g ml^−1^ of fibronectin (Asahi Techno Glass, Tokyo, Japan) for 30 min at room temperature and air-dried. The lower part of the chamber was filled with 10 *μ*g ml^−1^ of fibronectin in serum-free DMEM containing 0.1% BSA or with a medium containing BSA only. Cells (2×10^4^ well^−1^) in 50 *μ*l of DMEM containing 0.1% BSA were applied to the upper part of the chamber and incubated for 3 h under 5% CO_2_ at 37°C. The upper side of the filter was scraped with a cotton swab. The filters were fixed with formalin and stained with haematoxylin. The number of cells on the lower side was counted. The value indicates the mean±s.e. for triplicate data representing three independent experiments.

### Adhesion assay on the tissue

An adhesion assay on kidney sections was performed according to the Stamper–Woodruff assay (Stamper and Woodruff, 1976). The kidneys were removed from nude mice and immediately frozen by liquid N_2_. Horizontal sections (10 *μ*m thick) used for one assay were prepared from the same tissues and mounted on glass slides. Mock-transfected CHO-B2 and *α*5B2 cells (1×10^6^ cells in 1 ml of DMEM supplemented with 10% FBS) were fluorescence-labelled by 3 *μ*l of 1 mM BCECF-AM DMSO solution (Wako Pure Chemical Industries, Ltd, Hokkaido, Japan) and incubated for 30 min at room temperature. Excess BCECF was washed off with PBS and the cells were resuspended in 10 mM HEPES (pH 7.4). Cells (1×10^5^ in 0.1 ml of HEPES) were preincubated with GRGDS or GRGES peptide (200 *μ*g ml^−1^) for 30 min at 4°C and applied onto tissue sections marked by a wax pen and incubated for 30 min at room temperature with moderate rotation (60 r.p.m.). Unbound cells were washed away with PBS and the sections were fixed with 3% glutaraldehyde in PBS. Cell nuclei were stained with propidium iodide. The sections were air-dried and the number of cells adhering to glomeruli were counted by confocal laser microscopy (LSM-GB200, OLYMPUS, Tokyo, Japan). The value indicates the mean±s.e. for 20 glomeruli randomly selected and representing three independent experiments.

### Statistical analysis

The *χ*^2^ test was used to determine the significance of the *in vivo* metastasis experiments. The statistical analysis of the adhesion assay was performed by Kruskal–Wallis ANOVA. A *P*<0.05 value was regarded as statistically significant.

## RESULTS

### Expression level of *α*5 subunit on each CHO cell

**Figure 1:**
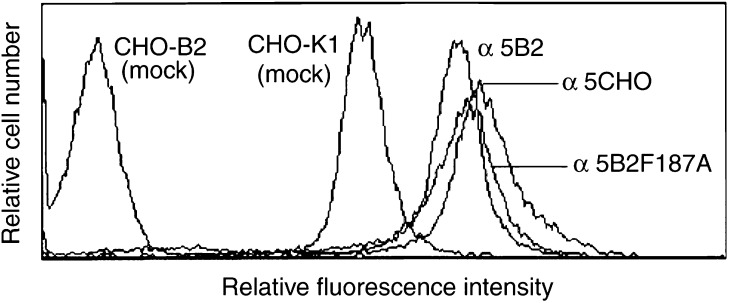
Flow cytometric analysis of *α*5 expression on CHO. Human and Chinese hamster *α*5 was detected by mAb KH72 on each CHO cell. CHO-K1 cells expressed *α*5 at the endogenous level and CHO-B2 cells, *α*5-defective mutant, did not express *α*5. Among three transfectants of *α*5B2, *α*5B2F187A, and *α*5CHO cells, *α*5CHO showed the highest expression of *α*5.

To confirm the expression level of the *α*5 subunit on each CHO cell type, we carried out a flow cytometric analysis (Figure 1

). Since the counterpart of the *α*5 subunit is only the *β*1 subunit, the expression level of the *α*5 subunit indicates that of *α*5*β*1 integrin. *α*5CHO cells showed the highest level of *α*5*β*1 integrin. *α*5B2 cells had a higher expression of *α*5*β*1 than CHO-K1 (mock) cells with the endogenous level. No *α*5 expression was observed in CHO-B2 (mock) cells, which are *α*5-defective mutants. *α*5 expression on *α*5B2F187A cells was almost the same level as *α*5B2 cells.

### Growth of primary tumour in vivo and cell proliferation *in vitro*

**Figure 2:**
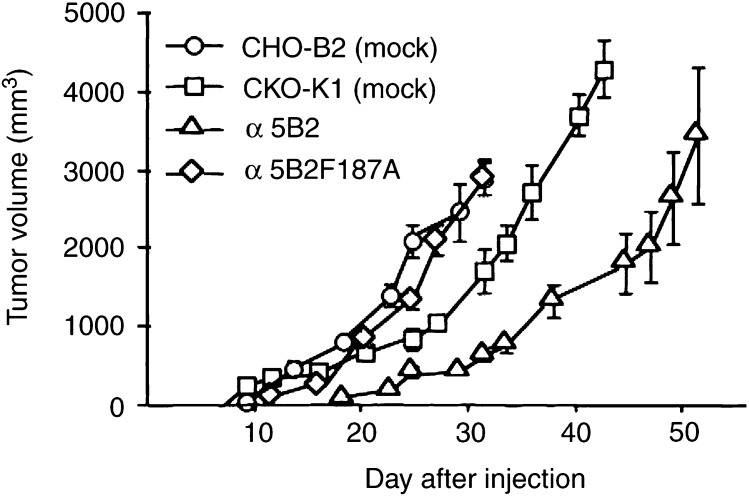
Growth of primary tumour in nude mice. Each growth curve shows primary tumour volume of nude mice inoculated subcutaneously with CHO cells (10^6^ cells mouse^−1^). The growth of tumour volume was reversely correlated with *α*5 expression level on CHO cells. In nude mice inoculated with *α*5CHO cells, no primary tumour was observed. Each value shows the mean±s.e. (*n*=8).

After the cells were inoculated subcutaneously into nude mice, the size of the tumour was measured twice a week (Figure 2

). The growth of the primary tumour was inversely correlated with *α*5 expression levels on CHO cells as previously reported (Schreiner *et al*, 1991). *α*5CHO cells expressing *α*5*β*1 at the highest level did not produce a primary tumour. In a cell-proliferation assay, CHO cells with increased *α*5 expression on fibronectin showed reduced cell proliferation as well as reduced cell growth *in vivo* (data not shown).

### Metastatic sites and rate of metastasis observed in nude mice subcutaneously inoculated with each CHO cell type

To identify the role of *α*5 expression on CHO cells in metastasis, we examined the organs with spontaneous metastasis in nude mice ((Table 1

**Table 1:** Metastasis formation of CHO and their transfectants inoculated subcutaneously into nude mice

**Cells and treatments**	**No. of mice analysed**	**% Lung**	**% Kidney**	**% Adrenal**	**% Lymph node**
CHO-B2 (mock)	21	90.4	0	19	9.5
CHO-K1 (mock)	22	100	22.7*	27.2	13.6
*α*5B2	19	89.4	47.3***	21	10.5
*α*5CHO	10	—	—	—	—
*α*5B2F187A	13	100	0	15.3	23
CHO-B2	23	91.3	0	21.7	8.6
CHO-K1	18	88.8	27.7*	27.7	11.1
*α*5B2 clone 1	10	100	30*	20	10
*α*5B2 clone2	10	90	50**	30	0
*α*5B2+GRGDS	20	90	10*	20	10
*α*5B2+GRGES	19	100	42.1	21	10.5

Mice were killed 7–8 weeks after inoculation. In metastasis inhibition experiment using GRGDS or GRGES peptide, 20 mg kg^−1^ peptide was administered to mice every other day since 5 days after inoculatin. Since *α*5CHO did not develop primary tumour, metastasis sites were not formed. The *χ*^2^ analysis shows that there is a significant difference compared with the metastasis rate CHO-B2

* *P*<0.05

** *P*<0.01

*** *P*<0.001).

The values of *α*5B2+GRGDS treatments were assessed by the *χ*^2^ analysis compared with that of *α*5B2+GRGES treatments.

). Lung metastasis was frequently seen in nude mice inoculated with parent CHO-K1 (mock, 100%), *α*5-defective mutants (CHO-B2, mock, 90.4%), or *α*5 transfectants (*α*5B2, 89.4%). This means that lung metastasis at least in this study appears to be primarily because of physical trapping of CHO cells by microvessels rather than interactions between *α*5*β*1 on CHO cells and fibronectin. Metastases in adrenal glands and lymph nodes were observed in nude mice at a lower level of 0–30%. These metastases also seem to have no relation to a change of *α*5 expressions on CHO cells. On the other hand, the rate of kidney metastasis changed in association with the expression levels of *α*5 on CHO cells. Both CHO-B2 cells with no *α*5 expression and *α*5B2F187A cells expressing mutated *α*5 did not give rise to kidney metastasis in nude mice. CHO-K1 cells with a native level of *α*5 produced kidney metastasis in nude mice at levels of 22.7–27.7%. *α*5B2 cells with a higher level of *α*5 than CHO-K1 cells caused kidney metastasis at a higher level of 30–50%. *α*5CHO cells with the highest level of *α*5 did not give rise to kidney metastasis in nude mice, since *α*5CHO cells could not produce a primary tumour because of their very slow growth. These findings indicate the possibility that kidney metastasis might be particularly caused by interactions between *α*5*β*1 on tumour cells and fibronectin in the kidney. Furthermore, kidney metastasis induced by *α*5B2 was significantly inhibited by intravenous administration of GRGDS peptide (10%) when compared to GRGES peptide (42.1%) (*P*<0.05), whereas the administration of GRGDS peptide did not affect the metastatic rate of any other organs investigated. This suggests that the adhesion of *α*5B2 cells to fibronectin via integrin *α*5*β*1 might have a crucial role in the induction of kidney metastasis.

### Macroscopic and microscopic findings of kidney metastasis in nude mice

**Figure 3:**
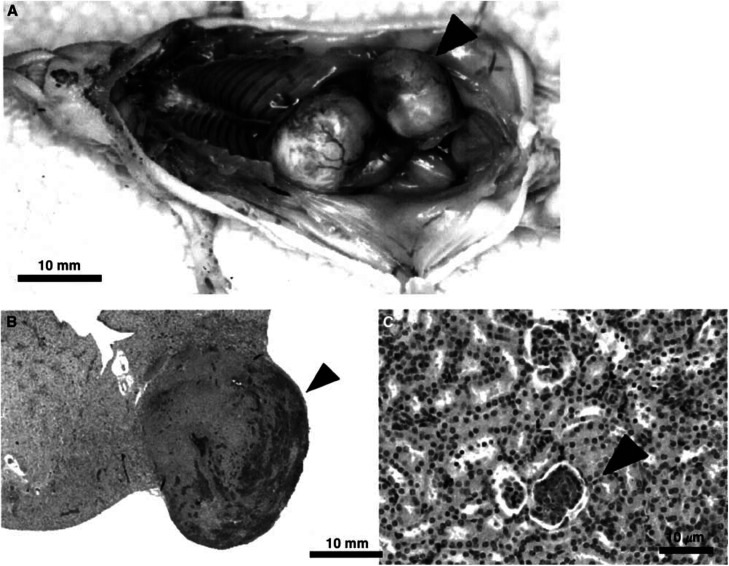
Macroscopic and microscopic findings of the kidney metastasis shown in a nude mouse inoculated with *α*5B2 cells. (**A**) a nude mouse with the kidney metastasis caused by *α*5B2 inoculation. The mouse was in supine position and intraperitoneal examination was performed after the laparotomy. An arrowhead shows the metastasis site in the right kidney; this mouse also has the adrenal metastasis. (**B**) Haematoxylin and eosin(HE)-stained section of tumour (arrowhead) in the kidney. All kidney metastases were found in the kidney cortex region. (**C**) HE-stained section of micrometastasis (arrowhead) developed in the kidney glomerulus. The lower glomerulus formed a micrometastasis site caused by *α*5B2 whereas the upper glomerulus was normal.

The macroscopic appearance of kidney metastasis is shown in Figure 3A

. The arrowhead indicates a metastatic site formed in the right kidney of a nude mouse inoculated with *α*5B2 cells, this mouse also has an adrenal metastasis. To identify where *α*5B2 cells were first trapped in the kidney, we observed micrometastatic sites in kidney sections stained with haematoxylin–eosin. In all nude mice with kidney metastasis, metastatic sites were formed in the kidney cortex region (Figure 3B, arrowhead). Furthermore, all micrometastases were shown in the kidney glomeruli (Figure 3C, arrowhead). These findings demonstrate that kidney metastasis develops first by the adhesion of *α*5*β*1 on CHO cells to fibronectin in the kidney glomerulus.

### Adhesion and migration of CHO cells to fibronectin *in vitro*

**Figure 4:**
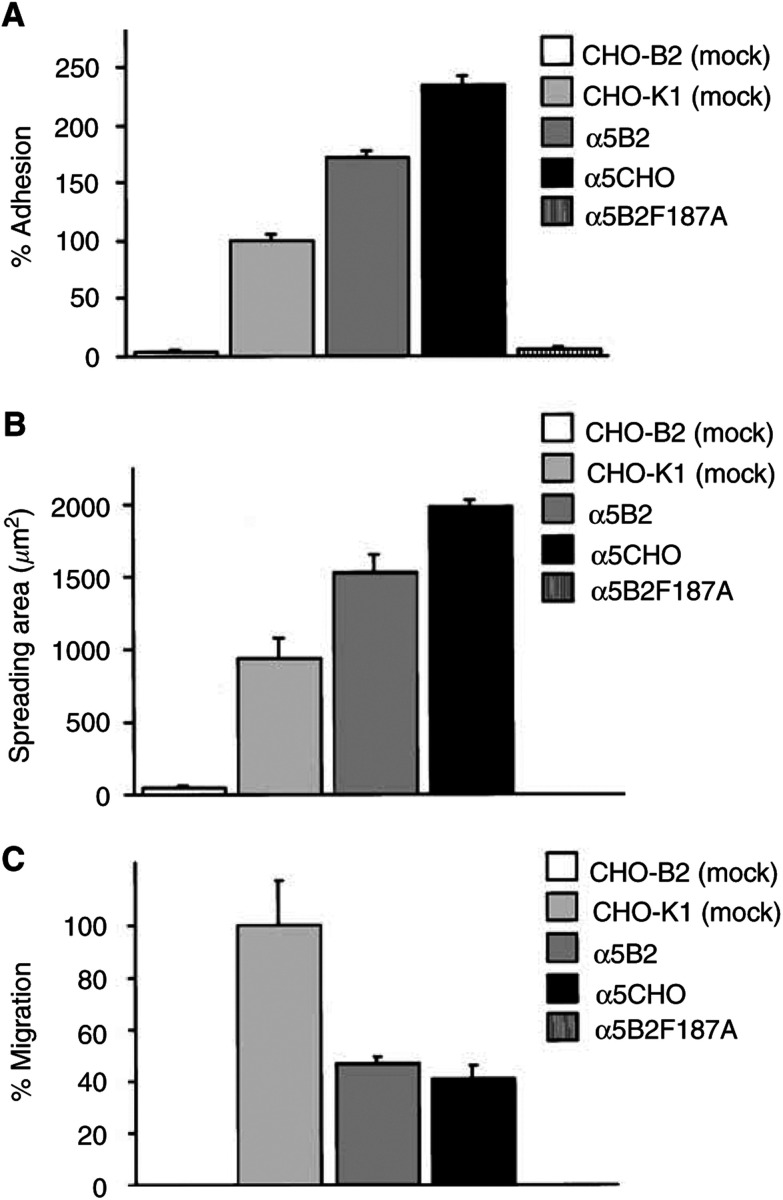
Adhesion and migration of CHO cells to fibronectin. (**A**) Adhesion assay demonstrating that adhesion activities to fibronectin in each CHO cell depend on *α*5 expression level. Cells (5×10^4^ well^−1^) in serum-free DMEM containing 0.1% BSA were plated on coated fibronectin(10 *μ*g ml^−1^) and incubated for 1 h at 37°C. Cells binding to fibronectin that were stained with crystal violet were assessed by measuring optical density at 550 nm. Results are expressed as the percentage compared with CHO-K1 cells (mock). Each value is the mean±s.e. of triplicate data representative for three independent experiments. (**B**) Spreading assay quantifying increased binding area in cell adhesion to fibronectin compared with cell spreading on BSA. Each cell in serum-free DMEM containing 0.1% BSA was plated on coated fibronectin (10 *μ*g ml^−1^) and incubated for 3 h at 37°C. Each value is the mean±s.e. of 20 cells randomly selected. The same results were obtained in two independent experiments. (**C**) Boyden chamber assay evaluating migration acitivity of each CHO to soluble fibronectin. A chamber with 10^5^ cells well^−1^ in the upper chamber and 10 *μ*g ml^−1^ fibronectin in the lower chamber was incubated for 3 h at 37°C in 10% CO_2_. The number of cells migrating to the lower side of the filter was assessed as a percentage compared with CHO-K1 cells (mock). Each value is the mean±s.e. of triplicate data, representative for three independent experiments.

To estimate whether it is cell adhesion or migration that is supposed to be crucial for kidney metastasis formation, the behaviour of each CHO cell type relative to fibronectin was analysed *in vitro*. In the adhesion assay, CHO cells with greater *α*5 expression effectively adhered to fibronectin, whereas *α*5B2F187A cells with the alanine mutation and CHO-B2 cells did not adhere to fibronectin (Figure 4A

). Likewise, CHO cells showed an increase in cell spreading that correlated with the overexpression of *α*5 (Figure 4B). Since tumour cells in intravasation penetrate into blood vessels containing abundant soluble fibronectin (300 *μ*g ml^−1^) in serum (Mosesson and Umfleet, 1970), the cell migration of each CHO cell type was estimated by a cell migration assay. In contrast to the results of adhesion and spreading assay, of all the CHO cells investigated, CHO-K1 cells with the native level of *α*5 migrated most effectively to fibronectin (Figure 4C). These data are consistent with the theory that cell migration is controlled by dynamic interactions between cell receptors and substratum ligands in a manner representing events at the front and rear of the migrating cells (Palecek *et al*, 1996, 1997; Regen and Horwitz, 1992). The rate of kidney metastasis appears to shift in parallel with the adhesion of CHO cells to fibronectin, except for *α*5CHO cells that do not develop a primary site.

### Adhesion of *α*5B2 cells to kidney tissue sections and inhibition by GRGDS peptide

**Figure 5:**
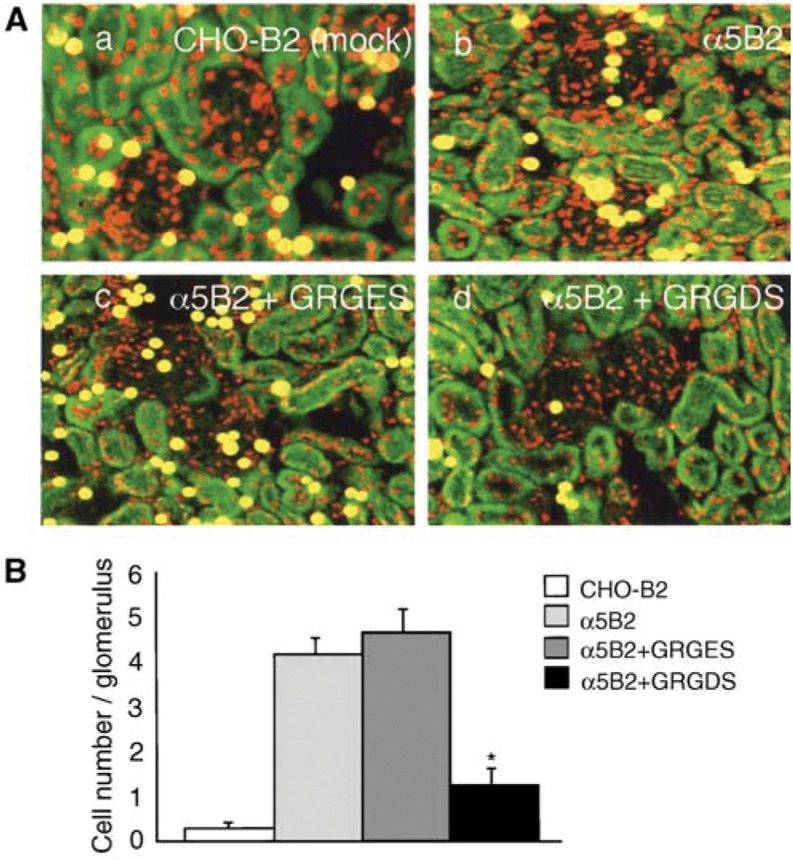
Effects of GRGDS peptide on adhesion of *α*5B2 to the glomerulus in the kidney section. (**A**) Laser microscopic appearance of adhesion assay on the kidney section. 10^5^ cells/section were incubated for 30 min with gentle rotation. a, CHO-B2 cells (yellow spots), *α*5-defective mutant, slightly adhere to the glomerulus (red spots). b, *α*5B2 cells which were *α*5 transfectants effectively adhered to the glomerulus. c, Adhesion of *α*5B2 cells to the glomerulus was not changed by administration of 200 *μ*g ml^−1^ GRGES peptide. d, Adhesion of *α*5B2 cells to the glomerulus was inhibited by administration of 200 *μ*g ml^−1^ GRGDS peptide. (**B**) Cell number per glomerulus (*n*=20). Note that adhesion of *α*5B2 cells with GRGDS treatment to the glomerulus was significantly inhibited as compared with GRGES treatment (^*^P<0.0001).

To identify whether kidney metastasis is primarily caused by interactions between *α*5*β*1 on CHO cells and fibronectin in glomeruli, we performed an adhesion assay with frozen sections. In this assay, the artificial binding of CHO cells to cortex regions such as uriniferous tubules other than glomeruli was seen because of adhesion of tumour cells in the absence of extravasation. Therefore, adhesion of *α*5B2 cells to glomeruli was estimated by focusing on the binding of cells (yellow spots) to glomeruli (accumulating red spots) (Figure 5A

). The number of cells on glomeruli for the different CHO cell types investigated is shown in Figure 5B. *α*5B2 cells adhered well to glomeruli whereas CHO-B2 cells with no *α*5*β*1 hardly adhered at all. The adhesion of *α*5B2 cells to glomeruli was significantly inhibited by the administration of GRGDS peptide, but not by GRGES peptide, when compared with the adhesion of *α*5B2 cells with no treatment (*P*<0.0001). These findings demonstrate that micrometastasis in glomeruli in mice may occur through interactions between *α*5*β*1 on tumour cells and fibronectin in the glomeruli.

## Discussion

In this study, we constructed CHO cell types with various levels of integrin *α*5*β*1 to investigate the relation between *α*5*β*1 expression on tumour cells and organ-preferential metastasis. Furthermore, we focused on alterations of the metastatic rate associated with different levels of *α*5*β*1. Previous studies have reported that the expression of *α*5*β*1 on tumour cells is inversely correlated with tumourigenicity (Giancotti and Ruoslahti, 1990; Schreiner *et al*, 1991). Moreover, the loss of fibronectin or *α*5*β*1 on tumour cells had no effect on tumourigenesis or metastasis in chimeric mice containing many *α*5-null cells (Taverna *et al*, 1998). However, it remains unknown how metastatic rates change in the case of abundant *α*5 expression on tumour cells. Furthermore, since the expression of *α*5*β*1 on tumour cells has been thought to make the phenotype of tumour cells normal, *α*5*β*1 expression might play a minor role in the tumour metastatic process. Here, we report that the expression of *α*5*β*1 on tumour cells is involved in the development of kidney metastasis. The inhibition of kidney metastasis *in vivo* and the adhesion of *α*5B2 cells to kidney sections *ex vivo* by administration of GRGDS peptide, both suggest that kidney metastasis might be induced by adhesion of tumour cells via *α*5*β*1 to fibronectin in the kidney glomerulus. Since RGD peptide is also found in laminin, collagen, and vitronectin, in addition to fibronectin, GRGDS peptide does not necessarily specifically inhibit the adhesion of *α*5*β*1 to fibronectin. CHO cells, however, predominantly express *α*5*β*1, and no ligand molecule other than fibronectin has been found in previous reports. Therefore, GRGDS peptide specifically inhibits the adhesion between *α*5*β*1 on CHO cells and fibronectin. Since a quarter of the total blood volume effused from the heart flows into the kidney (Churg and Sobin, 1982), many tumour cells seem to interact with capillaries in the glomeruli, suggesting the possibility that kidney metastasis could develop from tumour cells physically trapped by vessels. In this study, however, CHO-B2 cells with no *α*5 did not induce kidney metastasis in mice. Therefore, micrometastasis in glomeruli appears to be brought about primarily by the adhesive interaction of *α*5*β*1 on tumour cells with fibronectin in glomeruli.

In a spontaneous metastasis test, both CHO-B2 (mock) cells with no *α*5*β*1 and *α*5CHO cells with the highest level of *α*5*β*1 did not give rise to kidney metastasis in nude mice, whereas kidney metastases were seen in about 40% and 25% of mice inoculated with *α*5B2 and CHO-K1 cells, respectively (Table1). This demonstrates that tumour cells have integrin expression levels appropriate for developing metastatic sites. At a primary site, tumour cells with lower levels of *α*5*β*1 show rapid growth adequate for developing metastatic sites. However, once tumour cells intravasate, cells with higher levels of *α*5*β*1 appear to adhere easily to fibronectin to form metastatic sites.

How do tumour cells interact with fibronectin in kidney glomeruli? Since basement membranes of glomerular vessels contain a small amount of fibronectin compared with collagen and laminin (Yurchenco and Schittny, 1990) as well as general basement membranes, CHO cells with *α*5*β*1 might have difficulty in adhering directly to fibronectin in basement membranes of glomeruli during metastasis. The glomerular capillary is known to have a characteristic structure in relation to its surrounding basement membranes. In glomeruli, several blood vessels are surrounded by a basement membrane along the lateral side of endothelial cells, whereas a common blood vessel is covered with a basement membrane. Furthermore, at the medial side of the glomerular capillary, endothelial cells and mesangial cells without basement membranes are present (Ramzi *et al*, 1999). Since mesangial cells surrounded by glomerular blood vessels are known to produce ECM proteins containing abundant fibronectin (Couchman *et al*, 1994), tumour cells seem to interact easily with fibronectin by invading only the endothelial cells but not the basement membranes. On the other hand, lung, adrenal, and lymph node metastatic rates appear to be independent of *α*5 expression level on CHO cells. In these organs, integrins other than *α*5*β*1 or other molecules on tumour cells might be involved in the development of metastatic sites in addition to physical trapping of cells by microvessels.

In conclusion, we demonstrated that *α*5 expression levels on tumour cells might affect kidney metastasis rates. Therefore, the inhibition of kidney metastasis by targeting cell adhesion might be essential for metastasis therapy.
